# Coaggregation and biofilm growth of *Granulicatella* spp. with *Fusobacterium nucleatum* and *Aggregatibacter actinomycetemcomitans*

**DOI:** 10.1186/s12866-015-0439-z

**Published:** 2015-05-30

**Authors:** Maribasappa Karched, Radhika G. Bhardwaj, Sirkka E. Asikainen

**Affiliations:** General Facility Oral Microbiology Laboratory, Department of Bioclinical Sciences, Faculty of Dentistry, Kuwait University, Kuwait, Kuwait

**Keywords:** Coaggregation, Biofilm, *Granulicatella*, *Aggregatibacter actinomycetemcomitans*, *Fusobacterium nucleatum*, Autoaggregation

## Abstract

**Background:**

Members of fastidious *Granulicatella* and *Aggregatibacter* genera belong to normal oral flora bacteria that can cause serious infections, such as infective endocarditis. *Aggregatibacter actinomycetemcomitans* has long been implicated in aggressive periodontitis, whereas DNA-based methods only recently showed an association between *Granulicatella* spp. and dental diseases. As bacterial coaggregation is a key phenomenon in the development of oral and nonoral multispecies bacterial communities it would be of interest knowing coaggregation pattern of *Granulicatella* species with *A. actinomycetemcomitans* in comparison with the multipotent coaggregator *Fusobacterium nucleatum*.

The aim was to investigate coaggregation and biofilm formation of *Granulicatella elegans* and *Granulicatella adiacens* with *A. actinomycetemcomitans* and *F. nucleatum* strains.

**Results:**

*F. nucleatum* exhibited significantly (p < 0.05) higher autoaggregation than all other test species, followed by *A. actinomycetemcomitans* SA269 and *G. elegans. A. actinomycetemcomitans* CU1060 and *G. adiacens* did not autoaggregate. *G. elegans* with *F. nucleatum* exhibited significantly (p < 0.05) higher coaggregation than most others, but failed to grow as biofilm together or separately. With *F. nucleatum* as partner, *A. actinomycetemcomitans* strains SA269, a rough-colony wild-type strain, and CU1060, a spontaneous smooth-colony laboratory variant, and *G. adiacens* were the next in coaggregation efficiency. These dual species combinations also were able to grow as biofilms. While both *G. elegans* and *G. adiacens* coaggregated with *A. actinomycetemcomitans* strain SA269, but not with CU1060, they grew as biofilms with both *A. actinomycetemcomitans* strains.

**Conclusions:**

*G. elegans* failed to form biofilm with *F. nucleatum* despite the strongest coaggregation with it. The ability of *Granulicatella* spp. to coaggregate and/or form biofilms with *F. nucleatum* and *A. actinomycetemcomitans* strains suggests that *Granulicatella* spp. have the potential to integrate into dental plaque biofilms.

## Background

*Granulicatella adiacens* and *Granulicatella elegans* were previously known as “nutritionally variant streptococci (NVS)” [1]. The NVS group of bacteria were assigned an independent genus “Abiotrophia” [2] and later on, based on 16S rRNA sequence phylogeny, they were further divided into genera *Abiotrophia* and *Granulicatella* [3]. *Granulicatella* spp. are nonmotile, nonspore-forming, facultatively anaerobic Gram-positive cocci requiring complex nutrient-rich media for their growth. They are part of the normal oral flora [4,5], but similar to other oral species such as viridans streptococci and HACEK (*Haemophilus*, *Aggregatibacter*, *Cardiobacterium*, *Eikenella*, *Kingella*)-group of bacteria *Granulicatella* spp. can cause severe infections including infective endocarditis. Furthermore, most likely due to advancements in molecular biological methods, recent DNA-based studies have reported increased detection rates of *Granulicatella* spp. in periodontitis [6], caries [7] and endodontic infections [8,9].

Bacterial adhesion to surfaces is an important step in colonization and biofilm formation [10,11]. In the case of oral multispecies plaque biofilm development, early colonizer species adhere to nascent hard or soft tissues and provide a substratum for subsequent colonizers of the plaque biofilm. Essential for the development of multispecies biofilm communities is bacterial coaggregation, the adhesion of different bacterial species to each other. Furthermore, coaggregation is a key phenomenon that facilitates interaction among different bacterial species in the biofilm [12,13]. The interactions may occur between protein adhesins and polysaccharide receptors [14,15] or between proteinaceous adhesin-receptors [16].

*Fusobacterium nucleatum*, a Gram-negative obligate anaerobe in the oral cavity, plays a crucial role in the development and maturation of dental plaque biofilm due to its strong ability to coaggregate with early plaque colonizers, such as streptococci, and with the late colonizing Gram-negative anaerobes [15,17,18]. Apart this central role played by *F. nucleatum*, *P. gingivalis*, a late colonizer, also directly coaggregates with *Streptococcus mitis* [19], *Streptococcus oralis* [20], and *Actinomyces viscosus* [21]. Similarly, coaggregation between *Prevotella* spp. and *P. gingivalis* [22], and between *Tannerella forsythia*, streptococci and *P. gingivalis* [23] have been reported. Thus, it seems that in addition to using coaggregation as a mechanism of resistance to bacterial clearance, bacteria coaggregate with different oral species for specific additional benefits. For example, *S. gordonii* benefits from its coaggregation with *A. naeslundii* in arginine-deficient conditions through increase in the expression of arginine biosynthesis genes [24] and *A. naeslundii* also protects *S. gordonii* from H_2_O_2_-mediated oxidative damage [25]. Recently, it was suggested that by binding to streptococci, *F. nucleatum* overcomes resistance by oral microbiota and thus gets integrated into oral microbial community [26]. Further, adhesive capacity of *P. gingivalis* was reported to increase upon binding to *Treponema denticola* [27].

*G. adiacens, G. elegans* and *Aggregatibacter actinomycetemcomitans* are normal oral flora bacteria. While *Granulicatella* spp. belong to oral streptococci that bind to salivary pellicle on tooth surface, *A. actinomycetemcomitans* is a Gram-negative bacterium regarded as a late colonizer in dental plaque biofilm. Studies on *A. actinomycetemcomitans* interaction with oral streptococci have shown that H_2_O_2_ produced by *S. gordonii* enhances expression of the autotransporter ApiA, which leads to greater resistance against host’s immune response [28]. However, coaggregation between *Granulicatella* spp. and *A. actinomycetemcomitans* has not been studied. Investigating specific bacterial interactions may shed light on cooperation among these species of interest in biofilms. Therefore, our aim was to study coaggregation and biofilm formation of *Granulicatella* spp. with *A. actinomycetemcomitans*, a member of the HACEK group [29] in comparison with the “multipotent” coaggregator *F. nucleatum*.

## Methods

### Bacteria and culture conditions

*Granulicatella elegans* CCUG 38949 and *Granulicatella adiacens* CCUG 27809-T were cultured on chocolate blood agar with or without 0.001 % pyridoxal HCl [30] for 2 days at 37 °C and 5 % CO_2_ in air. *A. actinomycetemcomitans* strains were cultured on tryptic soy agar and incubated for 3 days at 37 °C and 5 % CO_2_ in air. *A. actinomycetemcomitans* SA269, a serotype d strain isolated from a 14-year-old female patient with localized aggressive periodontitis [31], was chosen to represent wild-type rough-colony strains which are fimbriated and strongly adhere to surfaces and form tenacious clumps in suspension [32]. *A. actinomycetemcomitans* CU1060 (a gift from D. Fine, Rutgers University, New Jersey, USA), a serotype f strain, is a spontaneous smooth-colony laboratory variant of the strain CU1000 isolated from a 13-year-old patient with localized aggressive periodontitis [33]. *Fusobacterium nucleatum* ssp. *polymorphum* NCTC 10562 (ATCC 10953) (isolated by H. Hoffman 1951; source “inflamed gingiva”) [34], was cultured on brucella blood agar containing 5 % defibrinated sheep blood and incubated at 37 °C for 2 days in anaerobic atmosphere (10 % H_2_, 5 % CO_2_, 85 % N_2_) using Anoxomat™ MarkII anaerobic gas filling system (Mart Microbiology, The Netherlands). Identities of the reference strains were confirmed by 16S rDNA sequencing as described earlier [35].

### Scanning electron microscopy

Agar blocks containing colonies of *Granulicatella* spp. were cut from the plates using sterile scalpels. For fixation, the blocks were immersed in PBS containing 3 % glutaraldehyde for 2 h on a rotator and then kept in a refrigerator overnight. After washing in PBS 3×, the agar blocks were incubated in 1 % osmium tetroxide for 2 h. The blocks were rinsed again as above and dehydrated in increasing concentrations of acetone from 30–100 %, 10 min in each on a rotator. The samples were then placed in a critical point dryer for complete drying, mounted on stubs by carbon double adhesive tape and finally coated with gold and stored in desiccator until observation. The samples were observed on Zeiss Leo SUPRA® 50VP field emission scanning electron microscope.

### Coaggregation assay

Previously published protocol was used with some modifications [36]. After the incubation, the bacterial colonies were suspended in sterile coaggregation buffer [10 mM Tris (pH 8.0), 1 mM CaCl_2_, 1 mM MgCl_2_, 150 mM NaCl]. Absorbance of the suspension was adjusted to an OD_600_ = 1. Five hundred microliters of cell suspensions from each of the test species and the partner species were added into a 1-ml cuvette. For autoaggregation, 1000-μl aliquots of each species were added into separate cuvettes. The cuvettes were incubated at room temperature and absorbance was read at 600 nm every 15 min for 2 h. Decline in optical density of the bacterial suspensions with time was used as a measure of autoaggregation or coaggregation. Percent coaggregation or autoaggregation [37] was calculated as follows:

$$ \%\ \mathrm{coaggregation}\ \mathrm{or}\ \mathrm{autoaggregation} = \left[\left(\mathrm{time}\ \mathrm{zero}\ \mathrm{value} - \mathrm{sample}\ \mathrm{value}\right)\ /\ \left(\mathrm{time}\ \mathrm{zero}\ \mathrm{value}\right)\right] \times 100 $$

### Phase contrast microscopy

Auto- and coaggregation of the test bacterial species were also studied by phase contrast microscopy. After preparing bacterial suspensions with or without partner species in coaggregation buffer as described above, 10 μl from each preparation was mounted on a microscopic glass slide with a coverslip and observed at 1000× magnification using phase contrast optics on Leica DMLM microscope.

### Biofilm growth

Static biofilm cultures were setup in 24-well cell culture plates using a previously established method [38]. Briefly, *G. elegans* CCUG 38949*, G. adiacens* CCUG 27809-T*, F. nucleatum* NCTC 10562 and *A. actinomycetemcomitans* strains SA269 and CU1060 were grown on their respective culture media as described above. Colonies were harvested, suspended in brucella broth and the cell suspensions adjusted to OD_600_ = 1 in brucella broth. A 100-μl aliquot from each strain separately or 50 μl from each of two partner strains was added into wells of a 24-well plate containing 900 μl brucella broth with 0.001 % pyridoxal. In parallel, a 20-μl aliquot from each bacterial stock suspension was streaked on respective growth media to ensure that the inocula used for biofilm culture were viable. Wells containing only broth served as negative control. After incubating for 3 days in anaerobic atmosphere at 37 °C, broth supernatant was removed and biofilms were washed once gently with sterile PBS. The plates were air-dried for 10 min at room temperature. One ml of 2 % crystal violet stain was added to each well and the plate was allowed to stay at room temperature for 10 min. The crystal violet stain was removed and the wells were washed 7–8 times with distilled water. To each well, 250 μl 95 % ethanol was added and the plate was incubated at room temperature on a shaker for 10 min. One hundred μl from each well was added into wells of a 96-well plate in duplicates and the absorbance was read at 590 nm using a microplate reader (Eon, BioTek® Instruments Inc., USA).

### Statistics

Mann-Whitney U test was used to compare groups. A p value of <0.05 was considered statistically significant. All experiments were performed in duplicates and 3 independent experiments were run.

## Results

### Pleomorphism of *Granulicatella* spp. cells

**Fig. 1:**
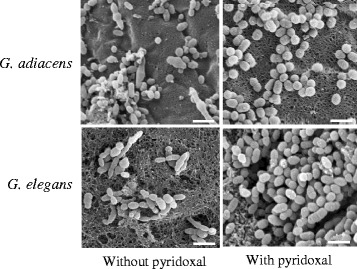
Pleomorphic cell morphology of *Granulicatella* spp. *G. elegans* CCUG 38949 and *G. adiacens* CCUG 27809-T were grown on chocolate blood agar supplemented with or without pyridoxal as described in the methods section. Scanning electron microscopy was performed as described in methods. The images were acquired at 10,000× magnification using Zeiss Leo SUPRA® 50VP scanning electron microscope. Bar represents 2 μm

As seen in scanning electron micrographs (Fig. 1), *G. elegans* and *G. adiacens* showed pleomorphic cell morphology when grown in the absence of pyridoxal supplement. The cells were elongated and often bulged in the middle. However, when the culture medium was supplemented with pyridoxal, both species assumed coccal shape. Further, Gram variability, which was evident when grown in the absence of pyridoxal, was no longer observed and the majority of the cells were Gram positive (data not shown).

### Autoaggregation

**Fig. 2:**
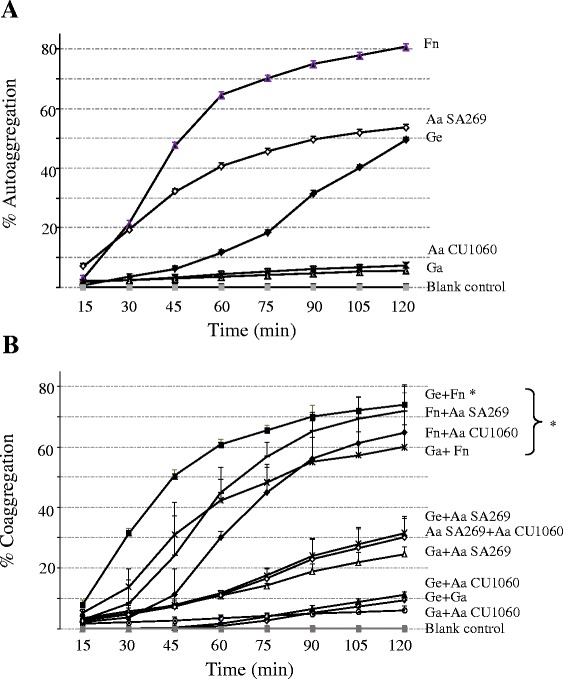
Autoaggregation and coaggregation of *Granulicatella* spp. Cell suspensions of each test bacterial strain separately or in combination with a partner species were prepared as described in the methods section. OD_600_ was measured every 15 min and the OD_600_ values were converted into percent autoaggregation (panel **A**) or coaggregation (panel **B**) using the formula described in the methods part. Abbreviations: Aa *A. actinomycetemcomitans*; Ge *G. elegans*; Ga *G. adiacens* and Fn *F. nucleatum*. The results are means (SD) from 3 independent experiments. *p < 0.05

**Fig. 3:**
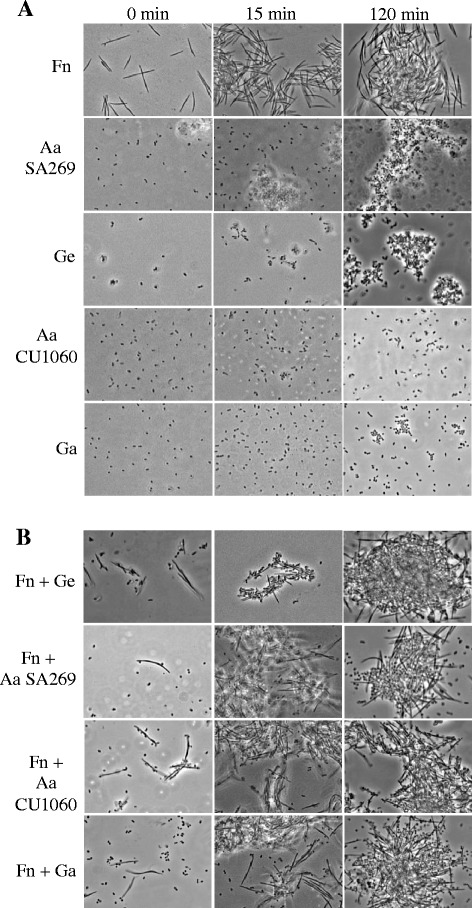
Auto- and coaggregation of *Granulicatella* spp. Bacterial cell suspensions of test species alone or in combination of the partner species were prepared as described in the methods section. A 10-μl aliquot from each suspension was applied on a glass slide with a cover slip and autoaggregation (panel **A**) and coaggregation (panel **B**) were assessed visually by observing at 1000× using phase contrast optics on a Leica DMLM microscope. Abbreviations: Fn *F. nucleatum,* Ge *G. elegans,* Ga *G. adiacens,* Aa *A. actinomycetemcomitans*

*F. nucleatum* was the strongest autoaggregating strain of all test species (Fig. 2A and Fig. 3A). It showed significantly (p < 0.05) higher percent of autoaggregation than all other test species at time points 30–120 min except *A. actinomycetemcomitans* SA269 at 15 and 30 min. Mean (SD) autoaggregation of *F. nucleatum* increased from 21.3 % (15 %) at 30 min to 48 % (14 %) at 45 min, 64.4 % (7.6 %) at 60 min and finally to 80 % (5 %) at 120 min. The next in autoaggregation efficiency was *A. actinomycetemcomitans* SA269, a rough-colony clinical isolate, which showed a mean (SD) autoaggregation of 19 % (7.5 %) at 30 min, 32 % (11.5 %) at 45 min and reached 53.6 % (10.4 %) at the end of the follow up. *G. elegans* began to autoaggregate after 45 min with a mean (SD) autoaggregation of 11.6 % (2.3 %), 18 % (4.9 %) at 75 min, 31 % (4 %) at 90 min finally reaching 49 % (1.4 %) at 120 min. *G. elegans* autoaggregation was significantly (p < 0.05) lower than that of *F. nucleatum* and *A. actinomycetemcomitans* SA269 at 45 min. However, at the end of the follow up, only *F. nucleatum*, but not *A. actinomycetemcomitans* SA269, showed significantly higher (p < 0.05) percent of autoaggregation than *G. elegans. G. adiacens* and *A. actinomycetemcomitans* CU1060, a smooth-colony variant, did not exhibit autoaggregation.

### Coaggregation

Overall, 3 distinct groups were observed based on coaggregation efficiency (Fig. 2B and Fig. 3B): 1) Strong coaggregators: *G. elegans* with *F. nucleatum*, *F. nucleatum* with *A. actinomycetemcomitans* SA269, *G. adiacens* with *F. nucleatum*, *F. nucleatum* with *A. actinomycetemcomitans* CU1060. 2) Moderate coaggregators: *G. elegans* with *A. actinomycetemcomitans* SA269, *A. actinomycetemcomitans* strains SA269 with CU1060 and *G. adiacens* with *A. actinomycetemcomitans* SA269. 3) Weak to Non-coaggregators: *G. elegans* with *A. actinomycetemcomitans* CU1060, *G. adiacens* with *A. actinomycetemcomitans* CU1060 and *G. elegans* with *G. adiacens*.

*G. elegans* together with *F. nucleatum* exhibited strongest (p < 0.05) coaggregation compared to all other species combinations. Mean (SD) coaggregation of *G. elegans* with *F. nucleatum* nearly doubled from 31.5 % (3.6 %) at 30 min to 60.6 % (2 %) at 60 min, finally reaching 74 % (3.5 %) at 120 min. Mean (SD) percent coaggregation of *G. elegans* with *F. nucleatum* was significantly (p < 0.05) higher [(31.5 % (3.6 %)] than that of *G. adiacens* with *F. nucleatum* [(13.7 % (5.6 %)] at 30 min. The difference was also significant (p < 0.05) at all time points starting from 30 min.

*F. nucleatum* in combination with *A. actinomycetemcomitans* SA269 showed mean (SD) percent coaggregation of 24 % (15.8 %) at 45 min, 44.7 % (7.6 %) at 60 min and finally increased to 71.8 % (7.2 %) at 120 min. The combination of *G. adiacens* and *F. nucleatum* showed mean (SD) percent coaggregation of 31 % (5.5 %) at 45 min, which increased to 60 % (6.6 %) at 120 min. *G. elegans* or *G. adiacens* exhibited moderate coaggregation with *A. actinomycetemcomitans* SA269 at later time points from 60 min to 120 min. However, neither the combinations of *G. elegans* or *G. adiacens* with *A. actinomycetemcomitans* CU1060 nor the combination of *G. elegans* and *G. adiacens* showed coaggregation within first 60 min, while a weak coaggregation of 6–10 % was evident at the end of the follow-up period.

Phase-contrast microscopy (Fig. 3) revealed that by 15 min, large numbers of cells of all partner species were bound to *F. nucleatum* cells. Additionally, numerous cells of partner species were also seen in close proximity to *F. nucleatum* cells. At the end of the follow-up period, i.e., 120 min, large clumps of *F. nucleatum* together with each of the partner species were abundantly present (Fig. 3B). Unlike *G. elegans* which was found to be binding to *F. nucleatum* cells already at 0 min, *G. adiacens* showed binding only at later time points, but not at 0 min. On the other hand, both *A. actinomycetemcomitans* strains, SA269 and CU1060, were seen bound to *F. nucleatum* at 0 min. Species combinations that included a partner species other than *F. nucleatum* are not shown as species were not distinguishable based on cell morphology.

### Biofilm formation

**Fig 4:**
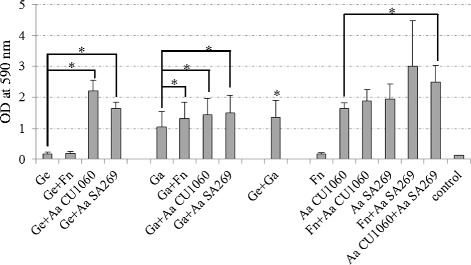
Biofilm formation by *Granulicatella* spp. in the presence or absence of *F. nucleatum* and *A. actinomycetemcomitans* strains. Bacterial strains were cultured separately or together with a partner species in 24-well plates in brucella broth in anaerobic atmosphere for 3 days as described in the methods section. After the incubation period, biofilms were washed gently to remove unattached cells and stained with 2 % crystal violet for quantifying biofilm mass. Abbreviations: Fn *F. nucleatum,* Ge *G elegans,* Ga *G. adiacens,* Aa *A. actinomycetemcomitans.* The results are means (SD) from 3 independent experiments. *p < 0.05

In the case of monospecies biofilm cultures, *A. actinomycetemcomitans* SA269 showed highest biofilm mass with a mean (SD) OD_590_ of 1.9 (0.5), followed by *A. actinomycetemcomitans* CU1060 and *G. adiacens,* which showed OD_590_ of 1.8 (0.3) and 1.04 (0.5), respectively, in the decreasing order (Fig. 4). OD_590_ values for *F. nucleatum* 0.17 (0.04) and *G. elegans* 0.16 (0.06) consistently showed poor biofilm formation ability in all three experiments. When *G. elegans* and *F. nucleatum* were grown as dual species biofilms, the biofilm mass did not increase compared to their respective monospecies cultures. In contrast, *G. adiacens* and *F. nucleatum* dual species biofilm exhibited a significant (p < 0.05) increase in biofilm mass compared to their monospecies cultures. Both *G. elegans* and *G. adiacens* showed significantly (p < 0.05) higher biofilm mass when grown together with either of the two *A. actinomycetemcomitans* strains. Dual species culture of *G. elegans* and *G. adiacens* showed significantly (p < 0.05) higher biofilm mass than their respective monospecies biofilm cultures did. In dual species biofilms, highest biofilm formation with a mean (SD) OD_590_ of 3 (1.5) was observed when *F. nucleatum* and *A. actinomycetemcomitans* SA269 were grown together. However, the biofilm mass was not significantly (p > 0.05) higher compared to *A. actinomycetemcomitans* SA269 cultured alone. Similarly, dual species culture of *F. nucleatum* and *A. actinomycetemcomitans* CU1060 did not show significantly (p > 0.05) higher biofilm mass compared to *A. actinomycetemcomitans* CU1060 cultured alone. When the *A. actinomycetemcomitans* strains SA269 and CU1060 were grown together, the biofilm mass was significantly (p < 0.05) higher than CU1060, but not SA269, cultured alone.

## Discussion

This study demonstrates that both *G. elegans* and *G. adiacens* have the ability to coaggregate with *F. nucleatum* and *A. actinomycetemcomitans*. These *Granulicatella* species also grew together with *A. actinomycetemcomitans* and *F. nucleatum* as dual species biofilms, except that *G. elegans*, despite being the strongest coaggregator with *F. nucleatum*, failed to grow as biofilm with this species.

Coaggregation efficiency was evaluated by a quantitative spectrophotometric method similar to several previous studies [39,40]. In this method, coaggregation is assessed by the decrease in the optical density of the bacterial suspension by time. The method allows definitive quantification of coaggregation in contrast to the visual scoring method used by several other studies [17,41,42]. Furthermore, we measured coaggregation for 120 min with 15 min time interval, which enabled us to investigate whether there were slow or late coaggregators among our test species. Varying incubation times from 1 to 30 min have been used for visual scoring of coaggregation [36,43]. Other methods of studying coaggregation among oral bacteria have been the use of radiolabelled [44,45] or fluorescently labeled bacteria [46]. Although these labeling based methods were claimed to be more sensitive, procedures like labeling of bacteria with fluorescent dyes and the need to pretreat cells in the case of certain dyes made them less simple compared to the spectrophotometric method used in this study. For biofilm studies, we used a standard static biofilm model, which was sufficient for direct quantification of biofilms by crystal violet staining.

Our interest towards *Granulicatella* species derived from recent findings that, besides systemic infections, granulicatellas also occur relatively frequently in dental infections [6-9]. The strains of *G. elegans* and *G. adiacens* we chose for this study were originally isolated from patients with endocarditis [47,48] and have been commonly used by medical researchers. On the other hand, the rationale for including *F. nucleatum* ssp. *polymorphum* (NCTC 10562) in this study was that it has been studied extensively for its strong coaggregation property and therefore known as a “bridge” organism between early- and late colonizing species during the process of plaque biofilm development. The *A. actinomycetemcomitans* strains selected for the present study were considered interesting, since both originated from young patients with aggressive periodontitis. However, the strains were of different serotypes and had dissimilar surface structures and autoaggregation behavior, as previously shown [32,33]. The strain SA269 (serotype d) is a representative of rough-colony, wild-type strains and strain CU1060 (serotype f) is a smooth-colony, non-fimbriated, spontaneous laboratory variant of the clinical strain CU1000 [33]. Using these *A. actinomycetemcomitans* strains we hoped to find differences in their behavior in the present experiments, which could be hypothesis generating in our further studies. As long known, *A. actinomycetemcomitans* is regarded as a late colonizer in dental plaque and coaggregates with other bacteria, e.g., *F. nucleatum* [43] and *P. gingivalis* [49] via serotype-specific polysaccharide antigen.

In our autoaggregation studies, *F. nucleatum* was the strongest autoaggregator followed by *A. actinomycetemcomitans* SA269 and *G. elegans*. Although, *F. nucleatum* has been extensively studied in the context of coaggregation, little is known about its autoaggregation [39,50,51]. Using a similar optical density method as in our study, *F. nucleatum* ATCC 25586, but not the strain *Fusobacterium nucleatum* ssp. *nucleatum* (ATCC 23726), was found to begin autoaggregating within 30 min, similar to our results; however, only in the presence of saliva [39], in contrast to our study where saliva was not required for autoaggregation. Thus, it seems that autoaggregation of *F. nucleatum* is strain-dependent and occurs via both saliva-dependent and -independent mechanisms. Autoaggregation of *A. actinomycetemcomitans* rough-colony isolates is well known and is mainly attributed to their long bundled fimbriae, while the smooth-colony strains are known to be non-autoaggregating [52,53].

In our study, the most efficient coaggregation, i.e., 45–65 %, was seen in the first 75 min in the case of “strong coaggregators” group in which *F. nucleatum* exhibited highest coaggregation with *G. elegans*. Majority of the earlier reports using either visual scoring or optical density method have demonstrated that *F. nucleatum* coaggregates strongly and rapidly (usually within 5–30 min) with most bacterial species in the oral cavity belonging to both early and late colonizer groups [45,54].

*G. elegans* and *F. nucleatum* exhibited strongest coaggregation, but failed to form biofilm together. This was unpredicted since coaggregation efficiency is thought to directly enhance biofilm formation in mixed species cultures [13]. It seems plausible that coaggregation alone was not adequate for a dual-species biofilm development by *G. elegans* and *F. nucleatum.* These species probably require presence of a third species for multispecies cooperation for successful biofilm formation. This has been demonstrated in a study where *F. nucleatum* did show some coaggregation but failed to grow as dual species biofilm with *Streptococcus oralis*, but when *Actinomyces naeslundii* was added, abundant biofilm growth was observed [55]. In that study, the authors concluded that *F. nucleatum* required *A. naeslundii* in the consortium to produce catalase to counter hydrogen peroxide produced by *Streptococcus oralis*. It should be remembered that in multispecies biofilms bacterial interactions are enormously complex and certain species exert antagonistic effect on others. For example, arginine deiminase produced by *Streptococcus cristatus* inhibited fimA expression in *P. gingivalis* [56]. Also, *Candida albicans* biofilm formation was inhibited by *A. actinomycetemcomitans* [57]. It seems that *A. actinomycetemcomitans* down-regulates expression of several proteins of other species in multispecies biofilm [58]. Thus, if the expression of a bacterial component essential for biofilm growth, e.g., quorum sensing signal molecules, such as autoinducer-2 [59], in *G. elegans* is down-regulated by the partner species, *G. elegans* might not be able to grow and persist in biofilm. Moreover, *G. elegans* might use coaggregation as a mechanism of cell-cell interaction to simply evade washout from saliva. It is likely that *G. elegans* requires presence of and interaction with other bacterial species in addition to *F. nucleatum* to successfully integrate itself into a dual- or multispecies biofilm.

In strong contrast to *G. elegans*, *G. adiacens* did not only coaggregate, but also grew together in biofilms with *F. nucleatum*. Both *G. elegans* and *G. adiacens* coaggregated with and grew as biofilms in the presence of either of the *A. actinomycetemcomitans* strains with the same efficiency. Our results confirm previous reports that *A. actinomycetemcomitans* coaggregates with *F. nucleatum* [36,43]. Importantly, both the fimbriated strain SA269 and the non-fimbriated strain CU1060 coaggregated with *F. nucleatum* with near-equal efficiency, suggesting that fimbriae did not contribute to coaggregation. In fact, previous studies have found that serotype-specific polysaccharide is responsible for coaggregation of *A. actinomycetemcomitans* with *F. nucleatum* [36,60] and *P. gingivalis* [49]. This is unlike autoaggregation of *A. actinomycetemcomitans,* which is solely attributed to fimbriae [32,53]. Furthermore, contradicting results have been reported regarding coaggregation of *A. actinomycetemcomitans* with *F. nucleatum*. While in one study *F. nucleatum* ATCC 10953 (equivalent of NCTC 10562 used in this study) did not coaggregate with any of the six *A. actinomycetemcomitans* serotypes [36], it did exhibit coaggregation with *A. actinomycetemcomitans* serotype b in several other studies [43,60]. Thus, no clear correlation has been established between *A. actinomycetemcomitans* serotypes and ability to coaggregate with other oral bacteria.

Since *F. nucleatum* and *A. actinomycetemcomitans* SA269 possess autoaggregation property, one might argue that coaggregation observed with their partner species was due to their autoaggregation. However, *G. elegans*, which did not show any autoaggregation during first 60 min, but not the strong autoaggregator *A. actinomycetemcomitans* SA269, showed strongest coaggregation with *F. nucleatum*. Further, *G. elegans* and *G. adiacens* showed only a weak coaggregation with the autoaggregating *A. actinomycetemcomitans* SA269 (Fig. 2B). Microscopic examination (Fig. 3B) of the coaggregates provided further evidence to specific coaggregation since partner species were seen avidly bound to *F. nucleatum*. Thus, our results clearly demonstrate that the observed coaggregation efficiencies were due to specific interaction between the partner species and not due to the autoaggregation property.

The ability of *G. elegans* and *G. adiacens* to coaggregate with and grow as biofilms together with *F. nucleatum* and *A. actinomycetemcomitans* is remarkable and suggests that they may form an important part of dental plaque biofilm. Further, since *Granulicatella* spp. belong to streptococci group of bacteria that are known to be the early colonizers of dental plaque, it was not surprising that *Granulicatella* spp. coaggregated with *F. nucleatum,* the so-called “bridge organism”. However, *Granulicatella* spp. may have additional benefits of this partnership. For example, if *Granulicatella* spp. lack β-lactamase, similar to some other oral streptococci [61], *F. nucleatum* producing the enzyme, may protect granulicatellas against β-lactam antibiotics.

## Conclusions

The ability of *G. adiacens* and *G. elegans* to coaggregate and form biofilms with *F. nucleatum* and *A. actinomycetemcomitans* probably offers granulicatellas additional benefits besides evasion from clearance in the oral cavity.
